# Thoracoscore and European Society Objective Score Fail to Predict Mortality in the UK

**DOI:** 10.14740/wjon897w

**Published:** 2015-02-14

**Authors:** Annabel Sharkey, Priyadharshanan Ariyaratnam, Vladimir Anikin, Elizabeth Belcher, Simon Kendall, Eric Lim, Babu Naidu, Wyn Parry, Mahmoud Loubani

**Affiliations:** a Department of Cardiothoracic Surgery, Castle Hill Hospital, Hull, HU16 5JQ, UK; b Department of Thoracic Surgery, Harefield Hospital Hill End Road, Harefield, Middlesex UB9 6JH, UK; c Department of Thoracic Surgery, John Radcliffe Hospital, Oxford, OX3 9DU, UK; d Department of Cardiothoracic Surgery, The James Cook University Hospital, Great Ayton, TS9 6BJ, UK; e Department of Thoracic Surgery, Royal Brompton Hospital, Sydney Street, SW3 6NP, UK; f Department of Thoracic Surgery, Heartlands Hospital, Birmingham, B9 5SS, UK; g Norfolk and Norwich University Hospital Thoracic Surgical Unit, Colney Lane, Norwich, NR4 7UY, UK

**Keywords:** Thoracoscore, ESOS, Risk scoring, Thoracic surgery, Lung resection

## Abstract

**Background:**

Thoracoscore and the European Society Objective Score (ESOS.01) are two scoring systems used in thoracic surgery to estimate operative mortality risk. We aimed to evaluate if these are valid tools for use in the UK population.

**Methods:**

A multi-center, prospective study was carried out on patients undergoing lung resection at six UK centers. Data were submitted electronically using our online data collection tool. Data were analyzed to determine the factors affecting mortality. A receiver operating characteristic analysis determined the ability of the thoracoscore and ESOS.01 to predict in-hospital mortality.

**Results:**

Data were complete for 2,245 patients. The observed in-hospital mortality was 31 patients (1.38%). Mean thoracoscore was 2.66 (SD ± 3.21). Gender (P = 0.004, hazard ratio 4.786) and co-morbidity score (P = 0.005, hazard ratio 3.289) were identified as risk factors for mortality. A sub-analysis was performed using data from 1,912 patients with complete data for ESOS.01. In this group, mean thoracoscore was 2.55 (SD ± 2.94), mean ESOS.01 was 2.11(SD ± 1.41), and these were statistically significantly different (P < 0.0001). The observed in-hospital mortality was 28 patients (1.46%). The c-index for thoracoscore was 0.705, and for ESOS.01 was 0.739.

**Conclusions:**

Both thoracoscore and ESOS.01 overestimated mortality in the UK population. There is a continued need to develop an appropriate risk prediction system for the UK.

## Introduction

There are two mortality risk assessment tools currently widely employed in thoracic surgery in Europe and the United Kingdom, the thoracoscore and the European Society Objective Score (ESOS.01) [1, 2]. The accurate assessment of the risk of post-operative mortality forms an integral part of the informed consent process, and also allows surgeon and unit outcomes to be compared. In the UK, departmental mortality outcomes are soon to be published online for all surgical specialities, and specifically for lung cancer resections, to allow comparisons of outcome between units. Ideally risk adjusted mortality should be published, and thus an accurate risk scoring tool is required. In addition, the British Thoracic Society recommends the use of a risk scoring tool in the pre-operative workup prior to lung resection [3].

Thoracoscore was devised in 2006 using the French Thoracic Surgery Database of over 15,000 patients. It is a logistic derived model consisting of nine pre-operative and operative variables [1]. The model was shown to be reliable and accurate with a c-index of 0.85 for the training set and 0.86 for the test set. The correlation between the expected and observed number of deaths was 0.99. It was then externally validated using patients from the United States in 2007 and 2009, and was again shown to be reliable and accurate with a c-index of 0.95 [4, 5]. Thoracoscore was shown to be a strong independent predictor of in-hospital mortality (odds ratio 1.20, 95% CI: 1.15 - 1.25, P < 0.001) [5]. However, as yet it has not proved to be accurate in numerous European or UK studies [6].

The ESOS.01 was created by the European Society of Thoracic Surgery Thoracic Surgery Database Project. It consists of only two variables, predicated post-operative FEV1 and age [2]. ESOS.01 has been shown to be superior to thoracoscore in one UK study but this was a single center, single surgeon analysis [7].

We aimed to determine whether either the thoracoscore scoring system or the ESOS.01 was valid tool for use in the assessment of mortality risk for the United Kingdom population.

## Materials and Methods

### Patients

We performed a multi-center study of all patients undergoing lung resection at six thoracic surgical centers in the United Kingdom over a 1-year period from July 2011 to July 2012, on behalf of the UK Thoracic Surgery Research Collaborative. Ethical approval was obtained from the National Research Ethics Service Committee North West-Haydock Park. Data were submitted either electronically using our online data collection tool, or by uploading the centers’ database directly to our system, from Birmingham Heartlands Hospital, Castle Hill Hospital, Hull, James Cook University Hospital, Middlesbrough, Norfolk and Norwich University Hospital, Oxford University Hospital and The Royal Brompton and Harefield Hospitals.

Patients’ data were collected as part of routine clinical practice at each center. Date from the nine fields making up thoracoscore were collected and submitted: age, gender, priority of the procedure, disease category, associated co-morbidities, ASA score, dyspnea score, performance score, type of procedure performed, and in-hospital mortality. Post-operative predicted FEV1 and 30-day mortality were also submitted if available. Post-operative predicted FEV1 was calculated using the standard formula: ppo-FEV1 = (pre-opFEV1) × (number of segments remaining/number of total unobstructed segments).

### Statistical analysis

Univariate analysis with the Fisher exact test was initially performed to determine independent predictive factors for in-hospital mortality. Variables with a level of significance less than or equal to 0.05 in the univariate analysis were included in the multivariate analysis by logistic regression. Using the thoracoscore definitions, age was divided into three groups (< 55 years, 55 - 64 years, and ≥ 65 years), co-morbidity into three groups (0, 1 - 2, and ≥ 3), gender (male vs. female), ASA score (1 - 2 vs. ≥ 3), dyspnoea score (0 - 2 vs. ≥ 3), performance status (1 - 2 vs. ≥ 3), diagnosis group (malignant vs. otherwise), procedure class (pneumonectomy vs. other), and priority of surgery (elective vs. urgent or emergency).

Model discrimination was assessed by the area under the receiver operating characteristic curve. Calibration was assessed by the Hosmer-Lemeshow goodness-of-fit statistic, and correlation was assessed using a Pearson’s correlation test.

Discrete variables are expressed as percentages and continuous variables as mean and range. All statistical analyses were performed with SPSS version 19 (Chicago).

## Results

Data were submitted for 2,570 patients. Three hundred forty-five patients were subsequently excluded due to incomplete data fields leaving us unable to complete the thoracoscore calculation. Decisions regarding fitness for surgery and patient selection were made by the individual operating surgeons at each unit. Of the remaining 2,245 patients, 1,245 patients were male (55.5%), mean age 64.4 years (SD ± 12.9 years). Table 1 includes the results for the remaining eight thoracoscore variables.

**Table 1:** Demographics, Thoracoscore and ESOS.1 Variables

Thoracoscore fields	Mean ± SD or percentage (n)
Age (years)	
< 55	18.8% (423)
55 - 65	27.7% (621)
> 65	53.5% (1,201)
Gender (male)	55.5% (1,245)
ASA	
< 2	70.3% (1,579)
> 2	29.7% (666)
Performance status	
0	96.7% (2,172)
1	3.3% (73)
Dyspnoea score	0.13 ± 0.336
Priority of surgery	
Elective	97.3% (2,185)
Urgent/emergency	2.7% (60)
Procedure class	
Other	94.2% (2,215)
Pneumonectomy	5.8% (130)
Diagnosis group	
Benign	20.3% (455)
Malignant	79.7% (1,790)
Co-morbidity score	1.25 ± 0.651
Thoracoscore	2.66 ± 3.21
ESOS	2.1 ± 1.41 (sub-analysis of 1,912 patients)
In-hospital mortality	1.4% (31)

The observed in-hospital mortality was 31 patients (1.38%). The mean predicted in-hospital mortality using thoracoscore was 2.66% (SD ± 3.21) (P = 0.02). Mean thoracoscore for those that died was 4.01 (SD ± 3.43) and for those who survived was 2.64 (SD ± 3.21), and this was statistically significantly different (P < 0.001).

Univariate analysis identified gender (P = 0.003) and co-morbidity score (P ≤ 0.001) as risk factors for mortality. On multivariate analysis, they remained strong predictors of in-hospital mortality. The hazard ratio of dying as a result of being male was 4.786 (P = 0.004) and as a result of a high co-morbidity score was 3.289 (P = 0.005) (Table 2).

**Table 2:** Univariate and Multivariate Analyses of Thoracoscore Variables

	Univariate P value	Multivariate P value	Odds ratio	Confidence interval	
				Low	High
Age	0.060				
Gender (male)	0.003	0.004	4.746	1.629	13.828
ASA	0.80				
Performance status	0.993				
Dyspnoea score	0.603				
Priority	0.189				
Procedure class	0.098				
Diagnosis group	0.152				
CMS	0.000	0.005	3.289	1.433	7.549

**Figure 1:**
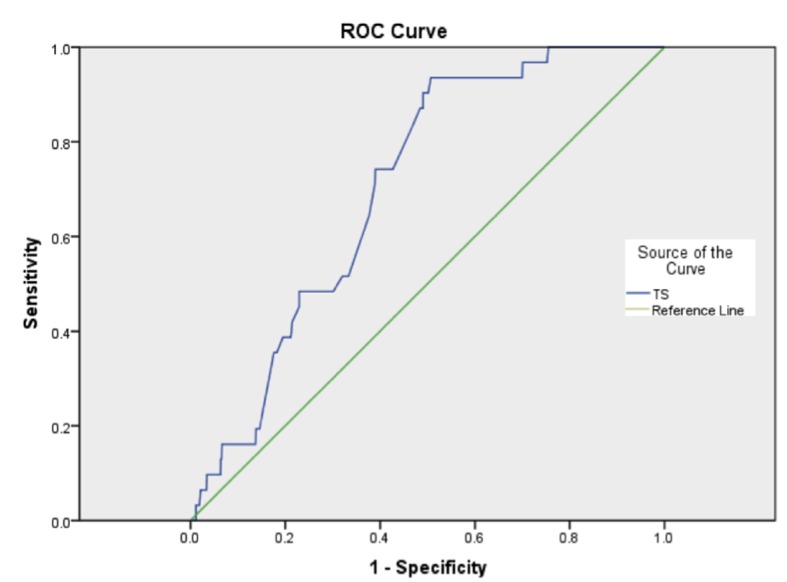
Receiver operating characteristic curve for thoracoscore (TS).

A receiver operator curve was produced (Fig. 1), and the c-index for in-hospital mortality and thoracoscore was found to be 0.705. This shows what at best might be an acceptable but by no means a good discriminatory ability, and was statistically significant (P < 0.001, 95% CI: 0.638 - 0.722).

We performed a sub-analysis using data from 1,912 patients to look at the predictive ability of the ESOS.01. In this group, mean thoracoscore was 2.55 (SD ± 2.94), mean ESOS.01 was 2.11 (SD ± 1.41), and these were statistically significantly different (P < 0.0001). The observed in-hospital mortality was 28 patients (1.46%). The c-index for thoracoscore was 0.738, and ESOS.01 was 0.739 (95% CI: 0.672 - 0.804 and 0.651 - 0.826 respectively).

**Figure 2:**
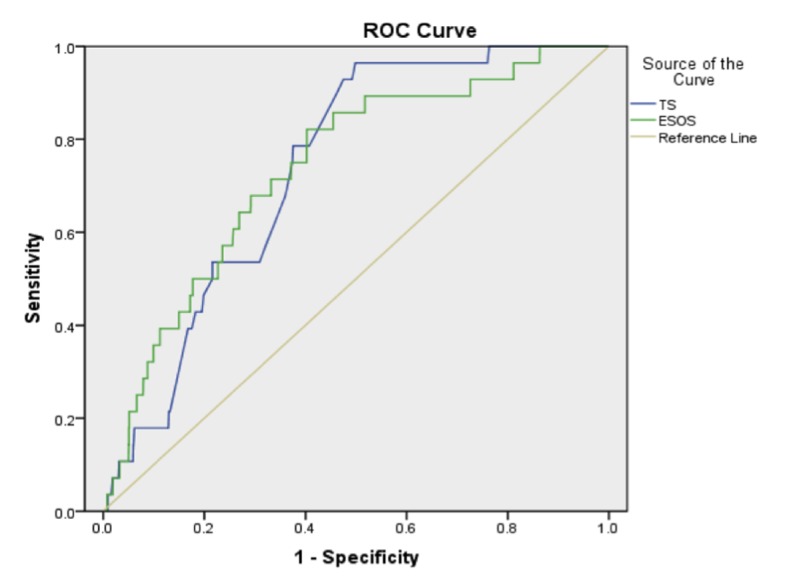
Receiver operating characteristic curve for thoracoscore (TS) and ESOS.01.

Despite an almost identical c-index (Fig. 2), there was poor correlation between the two scoring systems with a Pearson’s correlation co-efficient of 0.362.

## Discussion

An accurate assessment of in-hospital mortality risk is required in order to facilitate the consent process and to allow for risk adjusted surgeon and center-specific mortality comparisons to be made. A suitable surgical risk stratification tool must be able to discriminate between high and low risk patients within the applicable population, be calibrated, and also have “face validity” [8]. In the UK, we urgently required an accurate risk adjustment tool in thoracic surgery. Departmental, and ultimately surgeon-specific, mortality outcomes will soon be available in the public domain, allowing patients and commissioners, among others, to make direct comparisons between units. These outcomes need to be risk adjusted to prevent risk averse behavior from surgeons, given the potential for those units operating on high risk cases inaccurately being judged to be performing poorly if their outcomes fall below the level of others.

The thoracoscore was devised by Falcoz et al using the French Thoracic Surgery Database, using data from over 15,000 patients [1]. It is a logistic regression derived score comprising of nine pre-operative and operative variables. A subset of patients from this database were used to validate the scoring system, and it has also been further validated by a group using data from patients in the United States [4, 5]. It was shown to be accurate in both of these groups of patients. The ESOS.01 was devised by the European Society of Thoracic Surgery Thoracic Surgery Database Project [2]. Unlike thoracoscore, it consists of only two variables, age and post-operative predicted FEV1.

We sought to determine whether thoracoscore or ESOS.01 was accurate scoring system for use in the UK population. We chose not to include an assessment of the accuracy of the Society of Thoracic Surgeons’ scoring system in this study, given that it is not as widely used as the thoracoscore and ESOS.01 in the UK.

Using data from patients in six thoracic surgical centers around the UK, we calculated the thoracoscore for each patient and compared this with the observed in-hospital mortality. We then analyzed these data further to find whether any specific variables affected mortality. A sub-analysis of 1,912 patients was performed in order to determine the accuracy of the ESOS.01.

Mean thoracoscore was found to be 2.66%, almost double the observed mortality of 1.38%. However, mean thoracoscore for the patients who died was statistically significantly higher than those who survived, 4.01% versus 2.64% (P < 0.001). Only female gender and co-morbidity score were significant predictors of in-hospital mortality in this cohort. In the sub-analysis, mean thoracoscore was statistically significantly different to mean ESOS.01. Mean ESOS.01 was also significantly higher than the observed mortality, 2.11% vs. 1.46%, although this did not reach statistical significance.

Neither thoracoscore nor ESOS.01 was shown to be accurate at predicting in-hospital mortality. Both had a c-index of below 0.75 showing that they were inaccurate predictors of mortality in this group. The two scoring systems were also shown to be poorly correlated with one another.

The question to be asked from this multi-center study is, why are neither thoracoscore nor ESOS.01 accurate at predicting in-hospital mortality in the UK population? Many would argue that thoracoscore should be more accurate than ESOS.01, simply due to the higher number of variables included within the scoring system, but we have not seen this in this study. Although both scoring systems were devised using large numbers of patients, and thoracoscore has been externally validated a number of times, neither score has changed considerably since its initial creation. Our patient cohort is constantly evolving, as are the operative and peri-operative techniques that we employ. We are operating on older patients with more co-morbidities than we were 10 years ago, which should theoretically increase the operative risk. However we are also using more minimally invasive techniques and performing more limited anatomical resections, both of which have been shown to improve outcomes [9, 10]. Bearing all of this in mind, should thoracoscore and ESOS.01 not have evolved with us? We know from cardiac surgery and the use of the Euroscore that risk scoring systems need to be re-evaluated and altered as appropriate over time based on outcome data from large groups of patients [11].

Thoracoscore was devised using data from French patients, and validated using data from patients from the United States. We have shown that this scoring system is not accurate in the UK population. Were the patients used to devise the score different to those operated upon in the UK? The BTS guidelines for the radical treatment of lung cancer recommend using post-operative predicated FEV1 as a measure of fitness for surgery [3]. This and age are the only two factors used in ESOS.01, but it does not form part of the thoracoscore which begs the question, would a better scoring system combine all of these variables? The Liverpool group recently published a new risk scoring system based on a study using data from a single institution [12]. Their score, including factors such as age, gender and post-operative predicted FEV1, was shown in their patient group, to be more accurate than thoracoscore, ESOS.01 and the Society of Thoracic Surgeons model at predicting in-hospital mortality. Although this was a single center study, this gives further weight to the argument that the UK population may simply be different to that of France and the US, and therefore that we need to devise our own UK scoring system. In their paper they also identify another reason for the potential inaccuracy of the thoracoscore. It was devised for risk assessment for all thoracic procedures, be they for benign or malignant disease. A tool for all thoracic surgical procedures would be desirable, but given that in the UK mortality outcomes will be published for index procedures, based mainly around resections for malignant disease, we require a tool that is accurate for these procedures first and foremost. As they also rightly point out, there is no distinction between resections performed other than pneumonectomy versus other, and as they do not include post-operative predicated FEV1, there is no information regarding the potential effect of differing extents of lung resection on the patient. Does an open double sleeve lobectomy carry the same mortality risk as a video-assisted thoracoscopic wedge resection? This is a valid question to ask with regards further development of a new scoring system.

Powell et al recently provided insights from the National Lung Cancer Audit data, and derived a potential new scoring tool from their dataset of over 10,000 cases [13]. Although this study used an extremely large dataset, and their ability to do this should be commended, one must question both the accuracy of these data, especially given that over 60% lung function data were missing, and that ASA and MRC score were not included, plus the validity of utilizing 90-day mortality as the outcome measure. We have internally validated our data as compared with that returned to the National Lung Cancer Audit and found many discrepancies, particularly in timing and type of operation so the accuracy of their output must at least be considered when validating any risk scoring tools produced. It is conceivable that 90-day mortality may become the standard benchmark, but we feel that it is far more likely, and more useful in practice, for 30-day mortality to be used. A new risk scoring tool should be produced using all relevant variables, lung function and ASA score being particularly important for patients undergoing thoracic surgery, and not merely formed from the data available for the largest number of patients. It has been well described that outcome following thoracic surgery does not depend only on patient characteristics but on peri-operative factors [14]; however, any risk model used would be able to provide patients with a relative risk of surgery, assuming that all peri-operative care is standardized at least within single units. Furthermore, 90-day mortality would in some cases encompass the time in which adjuvant therapies are given, and therefore the risk models employing this time point cannot be accurate given the morbidity, and in some cases mortality, associated with these therapies. We appreciate that the size of this study is of the magnitude required to create a new scoring tool, but if we are to produce an suitable scoring system to aid patients in decision making, and to allow accurate monitoring of surgical outcomes, we must ensure that it is created using a large complete dataset including accurate demographic, staging and operative data, using the outcomes which are likely to be most relevant to patients and to the NHS as a whole.

We believe that the creation and validation of a new risk scoring system for patients undergoing lung resection, or at least a modification of these existing systems in line with our patient population and outcomes in the UK, is necessary. Ideally a new risk scoring tool would combine factors from thoracoscore and ESOS.01, as well as any others identified during the creating or adaptation process, as has been suggested in part by Powell et al [13]. Wider input of data is required to ensure its accuracy for the UK population. There is a marked variation in ethnicity around the UK and it has been shown in many studies that differences in race and ethnicity affect not only the receipt of cancer care but outcomes following treatment for several types of cancer [15, 16]. This study utilized data from patients from six different parts of England, but we require data from other areas of the UK, and of a magnitude similar if not exceeding that of the Powell study, to ensure any score we create is accurate, and based on data from a population as diverse as the UK itself. This risk adjustment tool must be appropriate for the UK population, and must be accurate at predicting mortality for patients undergoing lung resection. If we are to provide patients with an accurate risk of mortality from a thoracic operation, and if our outcomes are to be accurately risk adjusted to avoid risk averse behaviors, we must produce a risk scoring tool specific to the peri-operative period, a task without inherent obstacles given the multimodality nature of lung cancer surgery.
